# Identification, Recombinant Expression, and Characterization of LGH2, a Novel Antimicrobial Peptide of *Lactobacillus casei* HZ1

**DOI:** 10.3390/molecules23092246

**Published:** 2018-09-03

**Authors:** Junfang He, Xuegang Luo, Duxin Jin, Yunyang Wang, Tongcun Zhang

**Affiliations:** 1 Key Lab of Industrial Fermentation Microbiology of Ministry of Education & Tianjin Key Lab of Industrial Microbiology, College of Biotechnology, Tianjin University of Science and Technology, Tianjin 300457, China; HeJunfang2018@hotmail.com (J.H.); gogoxiaobai89@126.com (D.J.); WangYunyang1995@hotmail.com (Y.W.); 2 State Key Laboratory of Food Nutrition and Safety, Tianjin 300457, China

**Keywords:** antimicrobial peptide, *Lactobacillus casei*, *Staphylococcus aureus*, *Listeria monocytogenes*, pathogenic bacteria

## Abstract

*L. casei* HZ1 was identified from Chinese traditional fermented milk, and angiotensin converting enzyme inhibitory peptide was separated from its culture in our previous work. Here, LGH2 was a novel AMP, identified from the genome of *L. casei* HZ1. Altogether, roughly 52.76% of LGH2 was α-helical, with the remainder in β-strand and random coil in 50% TFE solution tested by CD. The peptide was also an amphipathic and cationic molecule, which was composed of 20 amino acid residues. The similarity of the amino acid sequence between LGH2 and Temporin-RN3 was highest. Then, the peptide successfully expressed in *E. coli* Rossetta (DE3) pLysS using the SUMO fusion expression system and purified by chromatography technologies. The molecular weight of the peptide was 2448 Da determined by MALDI-TOF MS. Antimicrobial tests showed that the peptide has strong activities against G+ bacteria, special for *S. aureus* (MIC = 4 μM). The toxicity assay showed that the peptide exhibits a low hemolytic activity against sheep red blood cells. The antimicrobial mechanisms of LGH2 against pathogens were further investigated by dye leakage, CLSM, SEM, and FCM assays. We found that LGH2 can bind to the cell membrane, and destroy its integrity. These significant results indicate that LGH2 has great potential to treat the infections caused by pathogenic bacteria such as *S. aureus*, and it provides a new template to improve antimicrobial peptides targeting antibiotic-resistant pathogenic bacteria.

## 1. Introduction

*Lactobacillus casei* (*L. casei*) is a kind of lactic acid bacteria (LAB), and is widely used in the food industry for its versatility, especially in fermented products. This species belongs to the genus *Lactobacillus* and is regarded as a kind of prebiotic. Many prebiotics are involved in the innate immune response because their metabolites regulate the host’s immune response and affect the gut’s immune system [1]. Natural host defense peptide, displayed antimicrobial activity against bacteria and fungi, associated with anti-inflammatory/immunomodulatory activity [2]. For example, the proline-rich peptide modulates the immune system of the wound-induced inflammation response via autocrine activity [3]. The important reason why *L. casei* can play such an important role in human health is that *L. casei* can produces a series of beneficial substances, such as antimicrobial peptides (AMPs), lactic acid, and exopolysaccharides. Especially, when it used to ferment milk, many beneficial substances were produced. As the main nutrient of milk, milk protein was also considered as containing important resources for peptides with antimicrobial activities [4]. Moreover, some antimicrobial peptides were released from milk protein after fermenting with LAB that can also have other physiological functions, such as immunostimulation and anticancer [5]. Among these beneficial substances, AMPs have attracted great interest for their special antibacterial properties and relationship with the immune response system [6,7].

At present, there is no unified theory to explain the antibacterial mechanism of AMPs. Generally speaking, most AMPs first interact with the bacterial cell membrane when they exert their antibacterial activities [8,9]. Membrane proteins and lipids of the cell are two main target substances for AMPs. Moreover, interference and permeation are the main antimicrobial action of AMPs, including barrel-stave, carpet, toroidal pore, and so on. Due to these reasons, AMPs interact directly with the cell membrane surface and can insert into the lipid bilayer, resulting in cell membrane disruption and leakage of cytoplasmic components [10,11].

AMPs are a kind of antimicrobial substance that exhibit a wide spectra of antimicrobial activities. Generally, AMPs are short polycationic peptides (<100 Aa), which are distributed widely in nature and can be secreted by bacteria, plants, animals, and human, and can be acquired from protolysate [12,13]. AMPs produced by Gram-positive bacteria have special inhibitory spectra and have been used as antimicrobial agents in various practical applications. In particular, the AMPs from LAB have been widely used as preservatives in the food industry, or as antibiotics in medicine [14]. In recent reports, scientists found that AMPs are important active substances for lactic acid bacteria in regulating intestinal microflora balance and health. In addition to antimicrobial activity, many AMPs also have anti-viral, anti-fungal, immunomodulatory, and anticancer activity [15,16,17]. At the same time, the resistance of pathogenic bacteria to AMPs has not been elucidated. Nowadays, AMPs are widely used as biopreservatives or antibiotic alternatives in the fields of medicine, agriculture, and the food industry. However, the activity of the peptide needs to be improved, and the toxicity of the AMP should be decreased. Many methods have been used to search for novel AMPs and to understand the relationship between peptide structure and antimicrobial activity, such as circular dichroism spectrum, scanning electron microscopy, magnetic resonance imaging, and so on. Furthermore, scientists can modify the peptide according to the characters of the novel peptides, to develop AMPs with high activity and low toxicity.

In our previous study, *L. casei* HZ1 was isolated from traditional Chinese fermented milk which was a common food in the daily diet of the Chinese. Earlier studies have revealed that *L. casei* HZ1 has high bile salt resistance and low cold and pH tolerance in vivo, and can produce angiotensin converting enzyme (ACE) inhibitory peptides during fermentation [18]. Meanwhile, the genome of *L. casei* HZ1was sequenced.

In this work, a novel AMP named LGH2 was identified from the genome of *L. casei*. Further, the AMP was expressed in *E. coli* and the antimicrobial activity and structure of the AMP was elucidated.

## 2. Results

### 2.1. AMPs Prediction

The genome of *L. casei* HZ1 has 2598 protein coding genes. We analyze all the proteins less than 10 KDa in molecular weight to find some novel potential AMPs. Further, the second structure and some physicochemical properties of AMPs were predicted using online software, such as molecular weight (MW) and PI by ExPASy, hemolytic characteristics by HemoPI, and Boman index (BI) and net charge (NC) by APD3. Finally, four AMPs with low toxicity, high structural stability, and good solubility were screened. After structure prediction using I-TASSER, we found that all AMPs have the potential to form the helix structure. The data was showed in the Table 1.

### 2.2. Antimicrobial Test of the AMPs

**Figure 1:**
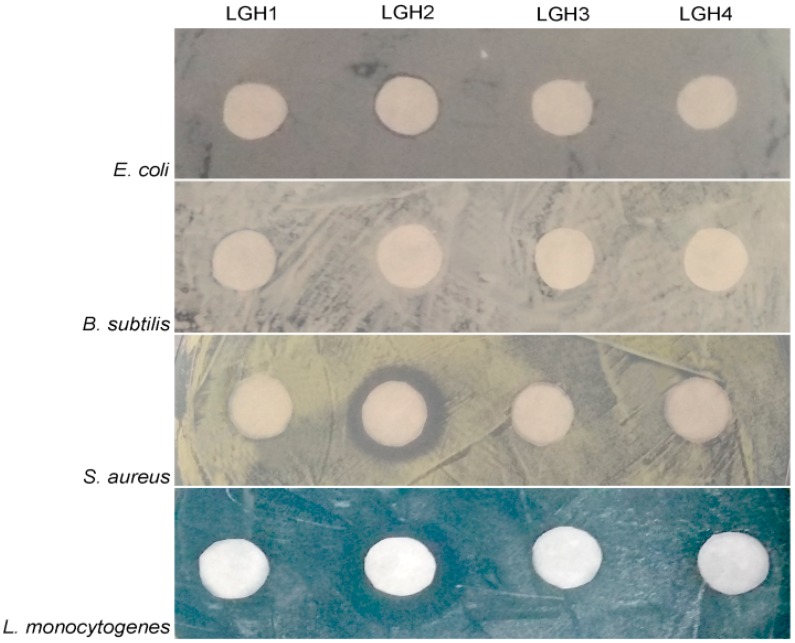
Antimicrobial test of four AMPs. The test operated at 128 µM. The four tested bacterial strains were *E. aerogenes* ATCC 13048, *L. monocytogenes* ATCC 7611, *S. aureus* ATCC 29213, and *B. subtilis* BL (collected by our lab).

First, the four putative AMPs were artificially synthesized and then the antimicrobial activities of the AMPs were tested using five typical bacterial pathogens. We found that only LGH2 has antimicrobial activity, the result is shown in Figure 1. The characterization data of four artificially synthesized AMPs are shown in Supplementary Materials Figures S1–S8.

### 2.3. Expression of the Lgh2 Fusion Protein in E. coli

**Figure 2:**
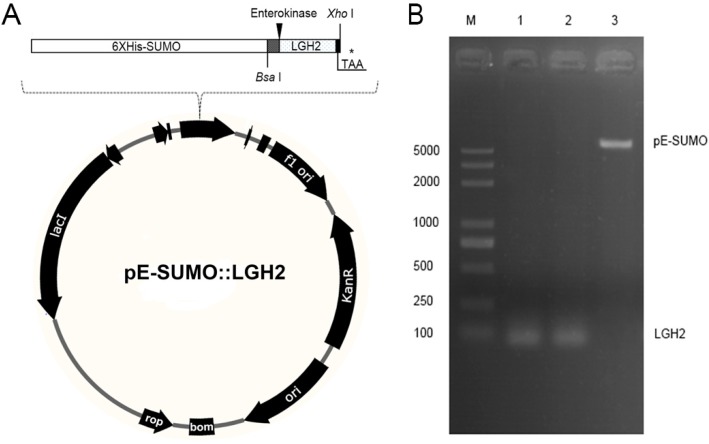
The construction of the expression vector. (**A**) Construction of pE-SUMO-LGH2 vector; * stands for termination codon. (**B**) Agarose gel electrophoresis of linearized pE-SUMO plasmid and *LGH2* gene. Lane M was the DNA marker; Lane 1 was the PCR product amplified from the T4-LGH2 vector; Lane 2 was the enzymatic production by both *Bsa* I and *Xho* I; Lane 3 was the lyzed pE-SUMO vector by both *Bsa* I and *Xho* I.

Because LGH2 is small and toxic to *E. coli*, it is difficult to express it directly in *E. coli*. We carried out a strategy to express LGH2 fused with a SUMO protein at the N-terminal end of the LGH2. The codon-optimized DNA sequence which encode LGH2 amino acid fragment was synthesized. Moreover, an enterokinase recognition site was designed at the upstream end of the LGH2 encoding fragment, in order to be cleaved by enterokinase. *Bsa* I and *Hind* III restriction endonucleases were added in the upstream and downstream of the sequence respectively, to make sure the fragment could be cloned into the pE-SUMO expression vector (Figure 2A). Agarose gel electrophoresis (Figure 2B) and DNA sequencing of the T7 region of the resulting pE-SUMO vector confirmed the successful integration of the LGH2 encoding fragment into the plasmid. The length of PCR production of LGH2 amplified from pE-SUMO-LGH2 recombinant vector was about 100 bps, which was similar to theoretical value of LGH2 fragment.

**Figure 3:**
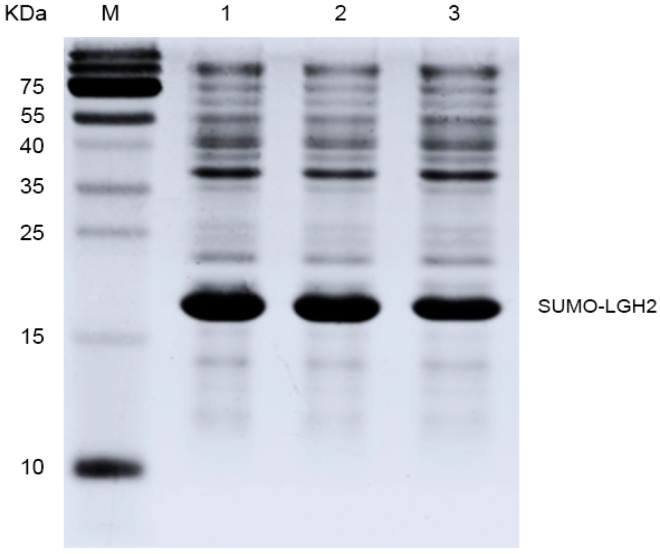
The expression of fusion protein SUMO-LGH2 from *E. coli* (DE3) plysS. Lane M contains the molecular mass marker; Lanes 1–3 are the protein expression of three monoclonal colons.

The prepared pE-SUMO-LGH2 vector was used to attempt to express the fusion protein in the host *E. coli* Rosetta (DE3) pLysS. The cell density was measured according to ultraviolet absorbance at 600 nm wavelength (OD600). When OD600 reached 0.6, the expression of the fusion protein was induced by IPTG. The expression of SUMO-LGH2 was analyzed by SDS-PAGE. As shown in Figure 3, the fusion protein was expressed successfully in the *E. coli*.

### 2.4. Purification and Analysis of LGH2

**Figure 4:**
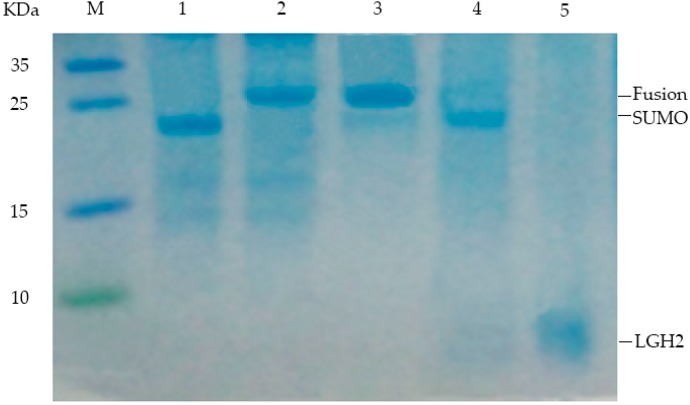
Analysis of expressed fusion protein and LGH1 by 16.5% Tricine-SDS–PAGE. Lane M, molecular mass marker; Lane 1, negative control; Lanes 2, colony induced by 0.5 mM IPTG; Lane 3, purified product of fusion protein isolated by Ni-Affinity column; Lane 4, production of fusion protein hydrolyzed by enterokinase; Lane 5, purification of LGH2 isolated by RP-HPLC.

After induction for 4 h, the cells were harvested and broken ultrasonically. The ultrasonic crushing mixture was separated by centrifugation. The supernatant was collected and the SUMO-LGH2 fusion protein was purified using Ni-NTA affinity column according to the operating manual. The purified protein was analyzed by Tricine-SDS-PAGE gel (Figure 4). The fusion protein has been isolated from the mixture successfully.

The isolated protein was desalinated by dialysis, and then cleaved by enterokinase after freeze-drying. After these processes, the peptide was obtained from the fusion protein. The peptide was also analyzed with a 16.5% Tricine-SDS-PAGE gel. From the Tricine-SDS-PAGE gel, the band of LGH2 was observed, and the fusion protein was cleaved sufficiently (Figure 4).

**Figure 5:**
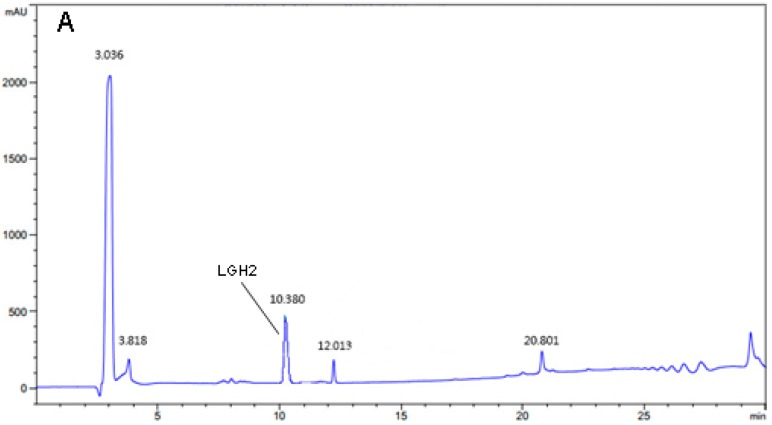

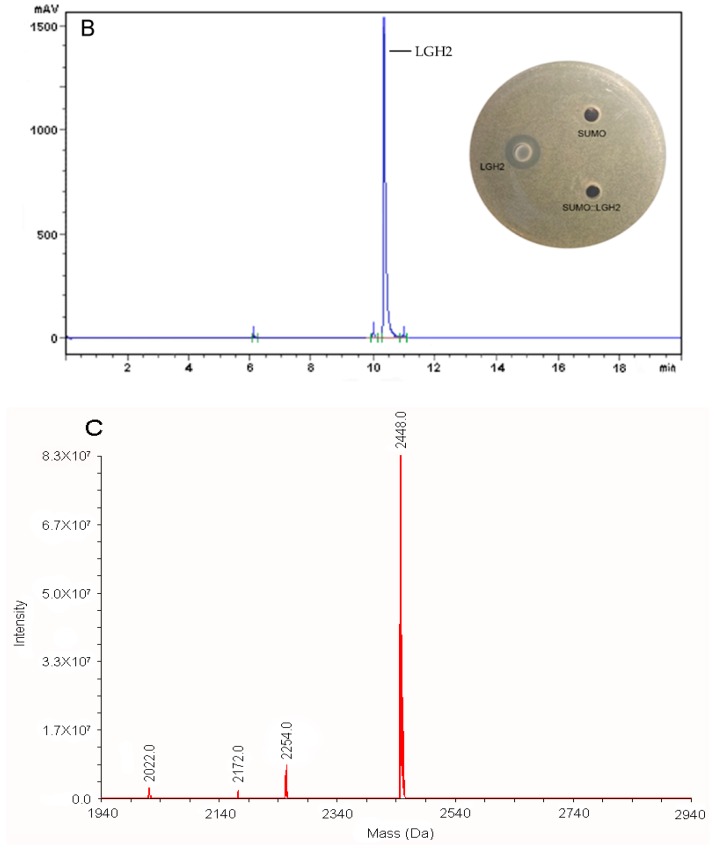
Purification and identification of LGH2. (**A**) Purification of hydrolysate by RP-HPLC. (**B**) Identification of purified peptide by RP-HPLC. The indictor strain was *S. aureus* ATCC 29213. (**C**) MALDI-TOF MS analysis of the purified LGH2.

The LGH2 was isolated and analyzed by RP-HPLC using a C18 reverse column. The result is shown in (Figure 5A,B). The elution peak appeared at approximately 10.38 min with the absorption wavelength of 220 nm, which was consistent with the standard sample. The liquid in the elution peak was manually harvested and dried by freeze-drying.

The dried LGH2 dissolved in water and analyzed by Matrix-assisted laser desorption/ionization-time-of-flight mass spectrometry to determine the identity of the molecule of the purified product. The result showed that the molecular weight of the expressed peptide LGH2 was 2448 Da (Figure 5C). This value is very similar to the theoretical value of molecular weight of LGH2, which is 2447.95 Da (Table 1).

### 2.5. Antimicrobial Spectrum of the LGH2

The MIC values of the LGH2 against pathogenic bacterium were tested. We found that only LGH2 has antimicrobial activity. The MIC values are listed in Table 2, which included two strains of Gram-negative bacteria, three strains of Gram-positive bacteria, and two strains of fungus. There was the best activity against *S. aureus*; the MIC value was only 4 µM. The MIC values of LGH2 against Gram-negative bacteria and Yeast fungi were higher than that of *S. aureus*.

### 2.6. Hemolysis Assays

**Figure 6:**
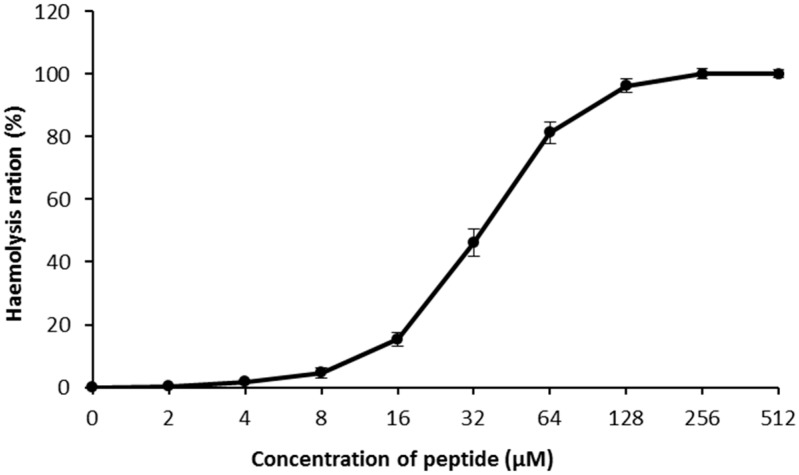
Hemolytic activity of LGH2. The experiment was performed in triplicate, for which the standard deviation is shown. The experimental values were normalized with the results for 1 × PBS (0% hemolysis) and 1% Triton X-100 (100% hemolysis).

In hemolysis assays, LGH2 showed a low toxicity against sheep blood red cells at 4 µM (Figure 6). The hemolytic process has a sharp rise from 8 µM to 128 µM, and finally a constant period of complete hemolysis. The assays confirmed that LGH2 had low hemolytic activity when the concentration was below 16 µM.

### 2.7. Effects of Recombinant LGH2 on the Cell Wall Integrity of S. aureus

**Figure 7:**
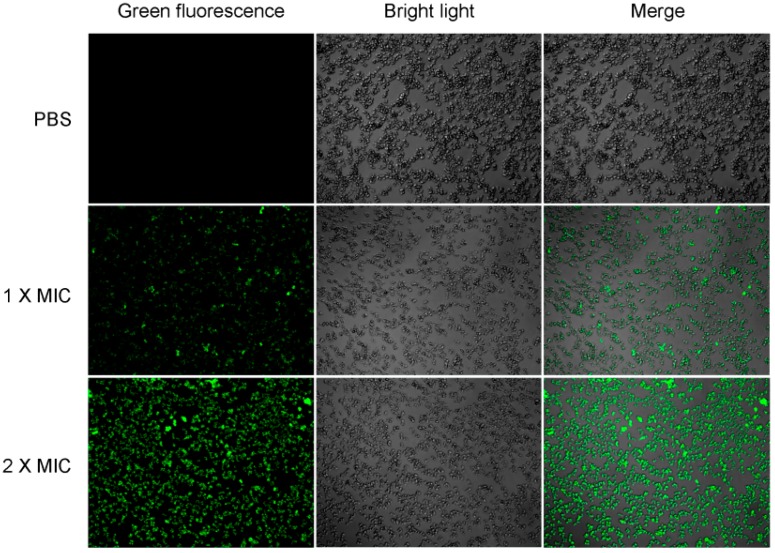
Binding of LHGH-1 to *S. aureus*. *S. aureus* was co-incultured with FITC-labeled LHGH-1 at PBS, 1 × MIC, 2 × MIC for 2 h and then examined by confocal laser scanning microscopy.

In here, *S. aureus* cells were cultured in LB liquid medium with FITC-labeled residue LGH2 (FITC-LGH2); we observed the relationship between LGH2 and bacterial cells using a laser confocal microscope (Figure 7). The fluorescence signal was observed from the cell membrane. This phenomenon means that LGH2 can bind to the cell membrane. When the dose of FITC-LGH2 increased, the intensity of the fluorescence signal also increased. According to this data, we suggested that the antimicrobial activity of LGH2 might depend on the dose. The characterization data of four artificially synthesized FITC-LGH2 were showed in Supplementary Materials Figures S9–S11.

**Figure 8:**
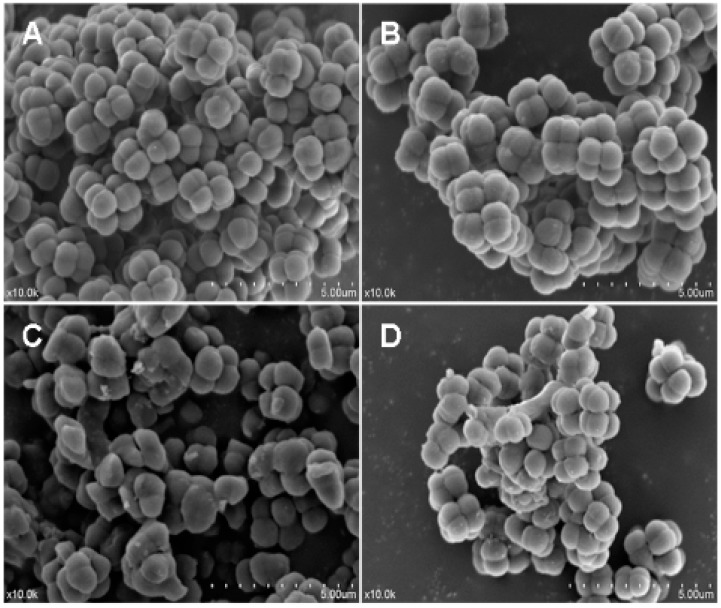
Morphological changes of *S. aureus* cells membrane by scanning electron microscopy. (**A**), (**B**), (**C**), and (**D**) are the image graphs of *S. aureus* cells treated with PBS buffer, LGH2 at 0.5 × MIC, LGH2 at 1 × MIC, and LGH2 at 2 × MIC, respectively.

The antimicrobial activity of LGH2 against *S. aureus* was tested by a Scanning electron microscopy assay (SEM). As shown in Figure 8, the control without peptides showed a smooth and intact surface (Figure 8A); in contrast, the peptide treatments induced significant membrane damage. The cytoplasmic membrane surfaces of peptide-exposed *S. aureus* cells appeared roughened, deformed, and covered by numerous blebs (Figure 8B–D). The recombinant LGH2 bound to the surface of *S. aureus* and caused cell shrinkage at the 1 × MIC.

**Figure 9:**
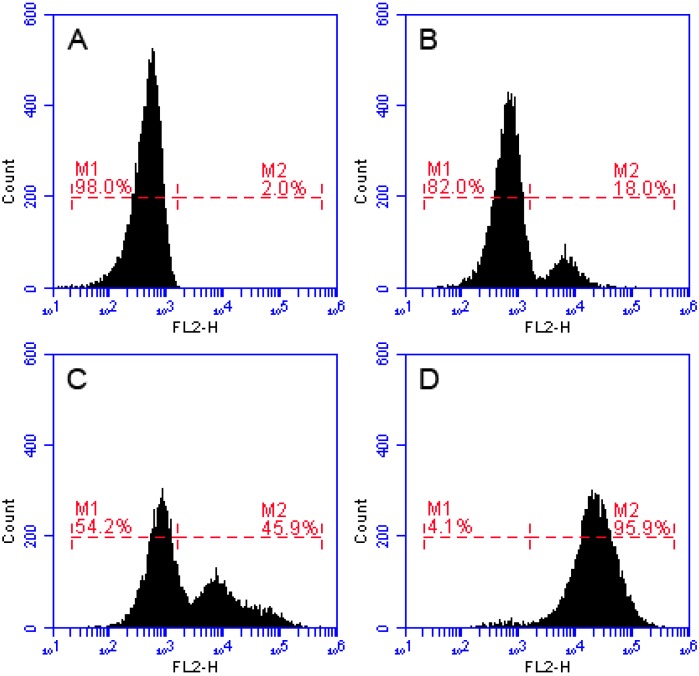
Flow cytometric analysis. The membrane damage of *S. aureus* treated by peptides was measured by an increase of fluorescent intensity of propidium iodide (PI) at 4 °C for 30 min. (**A**) No peptide, negative control; (**B**) LGH2 at 0.5 × MIC; (**C**) LGH2 at 1 × MIC; (**D**) LGH2 at 2 × MIC.

The method of FCM is well-established strategy to study the membrane disruption caused by AMPs. When the integrity of the cell membrane is damaged, the PI dye enters the cell and binds to the nucleic acid. Thus, cells with damaged cell membranes show a strong intensity of fluorescence [19,20,21]. In the FCM, we dealt *S. aureus* with PI nucleic acid dye, after 30 min of incubation of *S. aureus* with recombinant LGH2. Treated samples were analyzed by FCM. The dead cell count increased with processing dose. As shown in Figure 9, after treatment with 0.5 × MIC of LGH2 and 1 × MIC of LGH2, 18.0% and 45.9% of cells had PI fluorescent signals, respectively. More than 95.9% cells were labeled fluorescently after treatment with 2 × MIC of LGH2, while only 2.0% were labeled in the control group. The peptides possessed the ability to disrupt the cell membrane of *S. aureus* in a dose dependent manner.

## 3. Discussion

In the present study, bioinformatics analysis tools were used to explore potential AMPs from the genome of *L. casei* HZ1. Firstly, NCBI blast tools and CAMP_R3_ were used to identify possible areas of AMPs from the putative proteins (≤10 KD) in the genome. Then, we analyzed the characters of these putative AMPs. Some potential AMPs were further screened according to some key factors including helix conformation, net charge, molecular size, hydrophobic ratio, Boman index (protein-binding potential), hemolysis ratio, amino acid arrangement, and Isoelectric point. Finally, we identified four novel potential AMPs from the genome of *L. casei* HZ1.

Most AMPs interact with the bacterial cell membrane when exerting their antibacterial activities [8]. The peptides harboring a positive net charge are easier to interact with the negatively charged head groups of the phospholipids of the cell membrane [22]. From the physicochemical properties of these peptides, they all have a positive net charge (Table 1). Moreover, a higher positive Boman index is more advantageous to AMPs in binding with cell membrane proteins. Additionally, these peptides almost had a high hydrophobic rate; except LGH1 which was only 34%. This feature permits the peptides to form amphipathic conformations. When the peptides make contact with the cell membrane, the positive charged polar face helps them bind with phospholipids. Then, the nonpolar faces of AMPs insert into the membrane through hydrophobic interactions.

**Figure 10:**
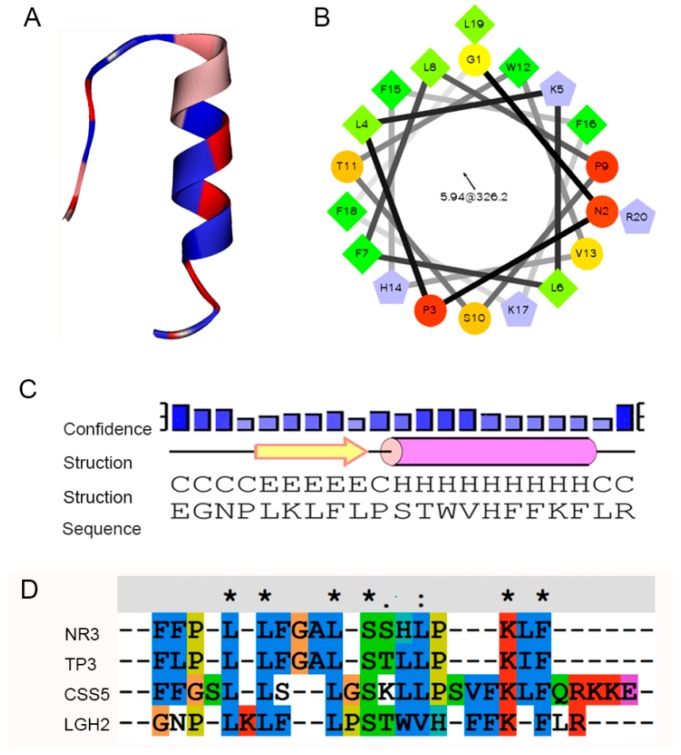
Bioinformatics analysis of LGH2. (**A**) Secondary structure of LGH2 (**B**) Helical wheel diagram for LGH2. (**C**) Secondary structure confidence analysis. (**D**) Multiple sequence alignment of LGH2 and its homologues by BLAST.

These peptides were artificially synthesized, and their activities against some typical microorganisms were analyzed. We found that LGH2 had antimicrobial activity, especially against *S. aureus*. However, the other three peptides did not display antimicrobial activity to the indicator bacteria. This result could be caused by the structure and composition of the peptides. Compared to the other three peptides, LGH2 has a higher net charge, a more hydrophobic rate, and a higher isoelectric point. Although LGH2 has a lower value in the Boman index and a lower molecular mass (Table 1), these characteristics were not the uppermost factors in determining antimicrobial activity. The hydrophobicity and the net positive charge are the two most important parameters that contribute to the membrane interaction [23]. The formation of α-helical structures improves the interaction of the AMPs with cell membranes and enhances the permeabilization of AMPS to the membrane [24]. The formation of LGH2 was advantageous to confirm the stability of the secondary structure of LGH2, which consisted of a α-helix conformation linked with β-strand conformations in the predicted secondary structure of LGH2 (Figure 10A). Meanwhile, the peptide has a distinguishable hydrophobic face and hydrophilic face (Figure 10B). These features are useful facilitators of antimicrobial activity. Moreover, the homology of LGH2 blasted in APD3 data. The results showed the highest homology between LGH2 and Temporin-RN3, which were separated from skin secretions of *Rana nigrovittata*. Both proteins had several similar fragments, such as L-X-L-F and L-X-S. These fragments were rich in the hydrophobic amino acids that are helpful to form helix conformations (Figure 10C).

**Figure 11:**
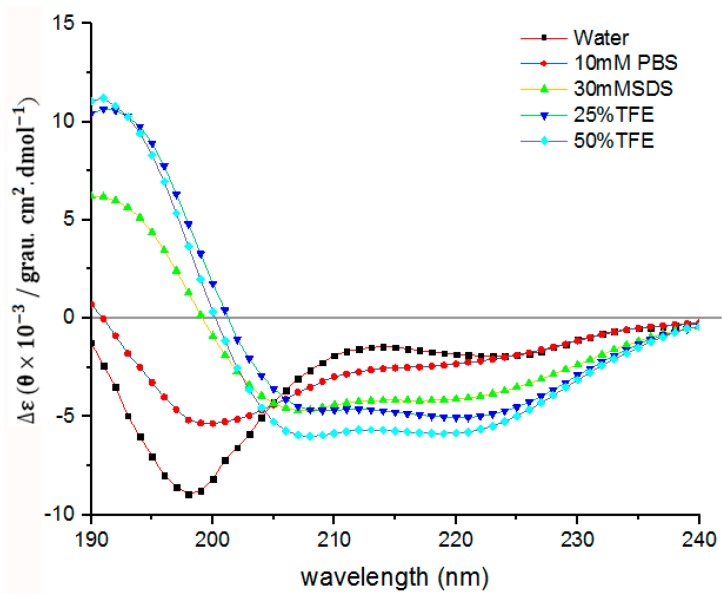
Circular dichroism spectra of LGH2. The peptides were dissolved in water, 10 mM sodium phosphate buffer (pH 7.4), 30 mM SDS, 25% TFE, and 50% TFE, respectively, to make the final concentration of the peptide 100 µM. The values from these three scans were averaged per sample.

In order to further understand the secondary structure of LGH2, we detected the secondary structure by CD. As shown in Figure 11, LGH2 had a stable α-helix conformation in 30 mM SDS, 25% TFE, and 50% TFE. They had negative peaks at 208 nm and 222nm, and a positive peak at 192 nm, these are typical characters of a α-helix conformation. However, the character of α-helix conformation of LGH2 was not obvious in water and 10 mM PBS. Moreover, the content of the conformation was calculated. LGH2 had 52.76% α-helix, when it was dissolved in 50% TFE buffer. But, the α-helix was only 4.35%, when LGH2 was dissolved in water (Table 3).

Although AMPs can be obtained from natural resources and chemical synthesis, gene engineering expression strategies are another good choice for AMP production, especially for peptides having pairs of disulfide bonds [25]. AMPs can be heterologously expressed in *Escherichia coli*, *lactic acid bacteria* (LAB), *Bacillus subtilis*, and yeast [26,27,28]. In bacterial heterologous expression systems, *E. coli* is a major host for heterologous expression of AMPs [29]. Compared with lactic acid, a bacteria host was often used in the food industry; *E. coli* is more widely applied in expression of proteins and peptides due to the excellent expression [30]. Because AMPs are usually small molecules and are toxic to the host cell, they cannot be expressed directly in the cells [25,31]. SUMO is a small protein which can enhance solubility of the fusion, so it is used in the expression of some small proteins or peptides [25,32]. In this study, LGH2 was effectively expressed and accumulated in the *E. coil* cell by SUMO fusion technology. Moreover, we used enterokinase to cut down the LGH2 from the fusion protein successfully. We used enterokinase because it can cleave the peptide from the fusion protein in the C-terminal of the identification sequence of Asp-Asp-Asp-Asp-Lys and its use makes sure no other amino acid residues exist in the N-terminal of the product. Finally, the antimicrobial activities of the expressed peptide were tested. The result showed that the MIC of LGH2 was 4 µM, when the MBC of LGH2 was 8 µM.

**Figure 12:**
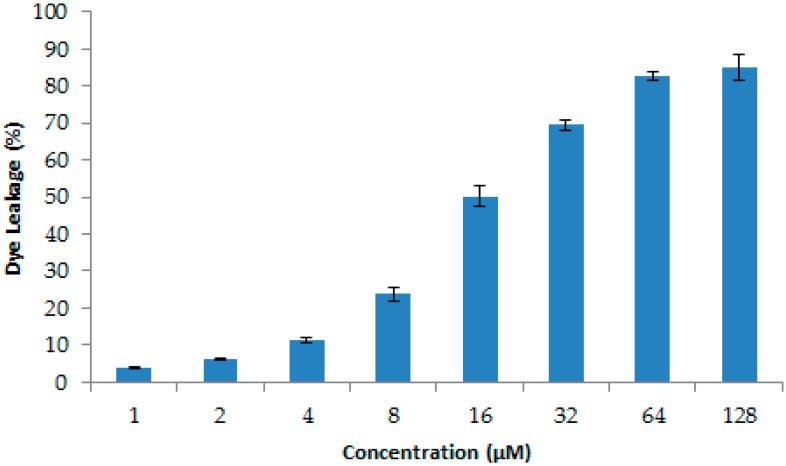
Release of calcein from CL/POPG (42:58) liposomes, with increasing concentration at 25 °C. Data are the average of at least three independent experiments± S.D. Error bars represent the S.D.

Nowadays, infections caused by resistant pathogenic bacteria are already playing a large part in hospital-acquired infections. *S. aureus* is a main pathogen for humans and animals, which can cause serious bacterial infectious diseases, such as pneumonia, enteritis, and ceratitis [33]. In this paper, we found that LGH2 has a special antimicrobial activity against *S. aureus*. Different from antibiotics, most AMPs inhibit pathogenic bacteria by interfering with the integrity of the cell membrane [34,35]. In order to understand the mode of LGH2 against *S. aureus*, we used the dye leakage assays CLSM, SEM, and FCM to analyze the mode of LGH2 against the Gram-positive bacterium *S. aureus*. In dye leakage assays because 1-palmitoyl-2-oleoyl-sn-glycero-3-phosphoglycerol (POPG) and cardiolipin (CL) were two the main cell membrane phospholipids of *S. aureus*, the calcein was entrapped in large unilamellarvesicles (LUV) composed of CL/POPG (42:58) [36]. The results are shown in Figure 12 below. The AMP of LGH2 could lead the LUV breakage, so that the calcein, which was entrapped in the LUV, escaped from the LUV. The rate of calcein leakage reached 85.13% when the concentration of LGH2 was 128 μM. Since lipids are the main components of cell membranes, the destruction of LGH2 to lipids could affect the integrity of the cell membrane [8].

In the CLSM, LGH2 can be bound to the cell membrane of *S. aureus*, and the fluorescence intensity of FITC increased with the dose of FITC-LGH2. In the SEM, the face of the cell became rough, shrinkage was observed, and cell debris were more and more present with increasing doses of LGH2. The leakage of cellular solutes resulted in the destruction of the cell membrane integrity. In FCM, the cell cytoplasmic membrane integrity was tested with PI dye. The result showed that cells dyed by PI accounted for 95.9% of total cells at 2 × MIC, while the control group was 2%. These results indicated that the activity of LGH2 against *S. aureus* depends on the dose and the lethality of LGH2 against *S. aureus* by destroying the cell cytoplasmic membrane integrity.

## 4. Materials and Methods

### 4.1. Bacterial Strains, Plasmid And Reagents

*E. coli* DH5α and *E. coli* Rosetta (DE3) pLysS were used for cloning and protein expression, respectively. *Staphylococcus aureus* (*S. aureus*, ATCC 29213), *Listeria monocytogenes* (ATCC 7644), *Klebsiella pneumoniae* (ATCC 700603), and *Enterobacter aerogenes* (ATCC 13048), was purchased from CMCC (Daxin, Beijing, China). *Lactobacillus casei* (*L. casei*) HZ1, *Saccharomyces cerevisiae* (*S. cerevisiae*), and *Pichia pastoris* (*P. pastoris*) were collected by our laboratory.

The pE-SUMO expression vector (Addgene, Cambridge, MA, USA); AMPs were synthesized by Bootech (Pudong, Shanghai, China); oligonucleotides were synthesized by Genewiz (Jiangbei, Nanjing, China); protein molecular weight markers were purchased from Sangon Biotech (Songjiang, Shanghai, China).

### 4.2. Identification of Potential AMPs from Genome of L. casei HZ1

All the proteins with less than 100 amino acid (aa) residues were selected from the genome of *L. casei* HZ1. The AMPs were evaluated by certain principles, as follows. (1) Containing a G-G/A leader motif or a signal peptide in the front of mature AMP [37,38]; (2) the sequences might form a helix; (3) the net charge of the sequence is positive; (4) protein-binding potential should not be less than 0 kcal/mol. All the dates were calculated by APD3 (http://aps.unmc.edu/AP/prediction/prediction_main.php), CAMP (http://www.camp.bicnirrh.res.in/prediction.php), and PeptideMass (http://web.expasy.org/ peptide_mass/) [39,40]. The second structure model was constructed by I-TASSER (https://zhanglab.ccmb. med.umich.edu/I-TASSER/) [41]. CellPPD was used to predict efficient cell penetrating peptides (http://crdd.osdd.net/raghava/cellppd/index.htmL) [42]. The hemolytic properties of peptides were forecasted by HemoPI online software (http://crdd.osdd.net/raghava/ hemopi/design.php) [43].

### 4.3. Construction of pE-SUMO-LGH2 Expression Vector

The gene flank with enterokinase recognition sites was synthetized by Genewiz (Jiangbei, Nanjing, China). The gene was digested by *Bsa* I and *Hind* III, and then ligated according to supplier’s instructions. The pE-SUMO-LGH2 vectors were transformed into *E. coli* DH5α competent cells by the standard CaCl_2_/heat-shock method. The recombinant pE-SUMO-LGH2 plasmids were sequenced using T7 forward primers.

### 4.4. Expression of the SUMO-LGH2 Fusion Protein 

The recombinant plasmid pE-SUMO-LGH2 was transformed into *E. coli* Rosetta (DE3) pLysS competent cells. The transformants carrying the correct gene, LGH2, were confirmed by PCR amplification using a pair of T7 primers and subsequent sequencing. Then the correct recombinant was incubated in LB broth (containing kanamycin of 50 mg/mL) and cultured overnight at 37 °C in LB-media. The next day, 1 mL of culture was injected into 100 mL fresh LB medium adding 1 g glucose, grown at 37 °C, and shaken at 200 rpm for 3 h until the value of OD600 was up to 0.6. IPTG was used to induce expression of the fusion protein. When the OD600 of cells reached approximately 0.6, 100 mg/mL IPTG solution was injected into the culture, in order to make the final concentration of the IPTG 1 mg/mL. When the IPTG was injected into the culture, the incubation temperature was changed to 25 °C. The cell was cultured at 220 r/min for another 4 h. Finally, the culture was centrifuged at 4000× *g* at 4 °C for 10 min, the cells were harvested and frozen at −80 °C.

### 4.5. Purification of LGH2 

The cells were resuspended in 10 mL of washing buffer (40 mM Tris-HCl; 500 mM NaCl, 15 mM imidazole, 8 M urea, pH 8.0) and washed three times with washing buffer before ultrasonic breakage. Ultrasonic breakage was conducted at 40 W for 20 min in ice-water. The broken cells were then centrifuged at 12,000× *g* at 4 °C for 15 min. Then, the centrifugal supernatant was collected and frozen in −80 °C.

Purification of SUMO-LGH2 fusion was purified by Ni-NTA under denaturing conditions. Briefly, the clarified supernatant flowed through the prepacked Ni-NTA agarose column at a flow rate of 1 mL/min. After that, the column was washed with five column volumes of binding buffer. The protein was eluted with three column volumes of elution buffer (40 mM Tris-HCl; 500 mM NaCl, 300 mM imidazole, 8 M urea, pH 8.0) from the column. The eluted liquid was collected, and the salt was removed by dialysis. The expression and purification of the recombinant protein was analyzed by 16.5% SDS-PAGE gel. The protein concentration was quantified by BCA protein quantification kit using normal rat IgG as a standard.

Subsequently, the lyophilized SUMO-LGH2 fusion protein was dissolved in enterokinase reaction buffer (50 mM Tris-HCl, pH 8.0, 1 mM CaCl_2_, 0.1% Tween-20). The SUMO-LGH2 fusion protein was digested by enterokinase at 37 °C for 24 h. The sample was dialyzed with distilled water and lyophilized again.

The lyophilized sample of the protein-peptide mixture was dissolved in initial mobile phase (acetonitrile: H_2_O = 10:90, containing 0.1% trifluoroacetic acid). The solution was centrifuged at 4 °C in 10,000× *g* for 20 min. The solution was analyzed by reverse-phase high-performance liquid chromatography (HPLC) (Agilent 1260, USA) with the C18 reverse phase chromatographic column (Kromasil-C18, 4.6 × 250 mm, 5.0 µm) at 30 °C. Eluent A was 1% TFA in water, eluent B was 0.1% TFA in acetonitrile. The sample eluted in a linear gradient of eluent B from 20% to 95% during 25 min with a constant flow rate of 1 mL/min. The detection wavelength was 220 nm. Fractions were manually collected according to absorption peak for next step analysis.

### 4.6. Matrix-Assisted Laser Desorption/Ionization-Time-of-Flight Massspectrometry Analysis

The predried peptide was dissolved in acetonitrile solution containing 0.1% TFA. The sample plate was inserted into the mass spectrometer and ionized by the short pulse laser. The Voyager time-of-flight mass spectrometer (AB Sciex, Framingham, MA, USA), equipped with 337 nm N2 laser and operated at an accelerating voltage of 20 kV. The spectrum after each laser pulse was summed to obtain qualitative information about the molecular weight of the compound.

### 4.7. Disk Diffusion Test

The activities of the four synthesis peptides were tested by disk diffusion test followed by Sun, Y., et al [44]. Agar plates were coated with *S. aureus*, *L. monocytogenes*, *E. coli*, and *B. subtilis*, and disks soaked with the four synthesis peptide were placed on the inoculated agar plates. The plates were incubated at 37 °C and the antimicrobial activity was assessed after 18 h by measuring the zones of inhibition in millimeters from each center.

### 4.8. Inhibitory Concentration Determination

The tested strains grew at 37 °C to an OD600nm of 0.4 in the medium. The minimal inhibitory concentrations (MIC) of the purified LGH2 were determined by the microtiter broth dilution method [45]. To define the MBC value, 5 µL of each clear well was cultured on solid media and incubated for another 24 h at 37 °C. The concentration at which no visible growth arose defined as MBC All experiments repeated three times independently.

### 4.9. Hemolytic Activity of LGH2

Hemolytic activity of the peptides was determined by measuring the release of hemoglobin from the sheep red blood cells [46]. Briefly, we centrifuged the whole sheep blood (Fisher Scientific, USA) at 400 g for 10 min at 4 °C to isolate erythrocytes. We washed erythrocytes with phosphate buffered saline (PBS) until the supernatant was clear and subsequently resuspended erythrocytes in PBS. 20 µL of serially diluted LGH2 added into 180 μL of erythrocyte suspension in a 96-well plate, and the final concentration of the peptide ranged from 0 µM to 512 µM. 1% Triton-X was used to determine 100% lysis. The plate was placed at 37 °C, we then centrifuged the cells treated with peptides for 2 h. We pipetted 20 µL of supernatant from each well and diluted it ten-fold with PBS. The absorbance of the solution measured at 567 nm.

### 4.10. Binding of LHH1 Fluorescently Labeled with Fluorescein

The logarithmic growth cells of *S. aureus* were separated and washed thrice with PBS at room temperature. The cells diluted to 10^6^ CFU/mL with PBS and co-incubated with the LGH2 labeled with fluorescein (FITC). The LGH2 fluorescently labeled with FITC according to standard procedures. The FITC-LGH2 was diluent with PBS to a final concentration of 1000 μg/mL. The initial cell density in the assays was 10^6^ cells per milliliter. Each assay was performed in duplicates. After 1 h of incubation with the FITC-LGH2, cells were harvested by centrifugation (1000× *g*, for 10 min) and visualized in an Confocal Laser Scanning Microscope equipped with a Zeiss Neofluor × 40 objective (numerical aperture 0.75).

### 4.11. Scanning Electron Microscopy Assay

The morphology of *S. aureus* treated with LGH2 was observed by SEM. The mid-logarithmic phase of *S. aureus* in a concentration of 1 × 10^9^ cells/mL was diluted with 200 µl of 0.01 M PBS and the culture was incubated with LGH2 for 1 h at 37 °C. The treated suspension was pelleted and washed with 0.1 M PBS at 4000 r/min for 10 min at 4 °C. Then, suspended in 2.5% glutaraldehyde and dried in air. Ethanol was used to wash the treated sample for dehydration. The dehydrated sample was sprinkled and imaged by Scanning Electron Microscope.

### 4.12. Flow Cytometry Assay

The bacterial cell membrane integrity was evaluated by flow cytometry (FCM) analysis [47]. In brief, *S. aureus* cultured in LB to mid-log phase, harvested, washed thrice with PBS, and then diluted to 10^6^ CFU/mL. The bacterial suspensions were incubated with the peptide (0.5 × MIC, 1 × MIC and 2 × MIC) for 30 min at 37 °C. The cells harvested by centrifugation, washed, and then incubated with 10 µg/mL propidium iodide (Sigma, Shanghai, China) for 30 min at 4 °C. After incubation, the unbound dye was removed by washing with PBS. The FCM instrument (Bio, Washington, DC, USA) was used to record the data with an excitation wavelength of 488 nm. Cells incubated with PI in the absence of peptide used as the negative control.

### 4.13. CD Analysis

The secondary structures of the peptides in different environments were evaluated using a MOS-450 circular dichromatograph (Bio-Logic, France). The spectra were recorded at a scanning speed of 10 nm/min at wavelengths ranging from 190 to 240 nm in sodium phosphate buffer (10 mM, pH 7.4), SDS buffer (30 mM, Sigma), or TFE (25%, 50% or 75%, Sigma, USA). An average of three scans were collected for each peptide. The final concentration of LGH2 was 100 μM. The acquired CD signal spectra were converted to the mean residue. Secondary structure of LHH was determined by deconvoluting CD data at Dichroweb platform (http://dichroweb.cryst.bbk.ac.uk/html/links.shtml) (Whitmore & Wallace, 2004).

### 4.14. Dye Leakage Assays

Calcein-entrapped large unilamellarvesicles composed of CL/POPG (42:58) were prepared as described by Rodrigo M. Verly., et al [36,48]. Briefly, CL/POPG lipids were dissolved in chloroform, dried with nitrogen gas and resuspended in dye buffer solution (10 mM HEPES, 50 mM calcein, pH 7.4). The suspension was subjected to 10 frozen-thaw cycles in liquid nitrogen and extruded more than twenty times through polycarbonate filters with two stacked 100 nm pore size filters. Untrapped calcein was removed by gel filtration on a Sephadex G-25 column. The leakage of calcein was detected by measuring the fluorescence intensity at an excitation wavelength of 485 nm and emission wavelength of 530 using an infinite M2 PRO microplate reader (TECAN, Melbourne VIC, Australia). The 100% of CF fluorescence was determined by 1% Triton X-100. The percentage of CF leakage was calculated by the below equation.

(1) $$\mathrm{Dye}\;\mathrm{release}\left(\%\right)=\frac{F-F_{0}}{F_{100}-F_{0}}\times 100\%$$

where *F*_0_ is the fluorescence intensity of liposomes without LGH2, *F* is the fluorescence intensity of liposomes added LGH2, and *F*_100_ is the fluorescence intensity of liposomes added 1% Triton X-100.

### 4.15. Statistical Analysis

All tests were carried out in triplicate. Statistical analyses were performed using IBM SPSS Statistics for Windows, Version 19.0. Data are presented as mean ± standard deviation (SD). The differences were considered to be statistically significant at *p* < 0.05.

## 5. Conclusions

In this work, a novel AMP (LGH2) was identified from the genome of the *L. casei* HZ1. This peptide had a higher antimicrobial activity against *S. aureus* than the other bacteria, and the MIC was 4 μM. Then, heterologous expression of the peptide fusion with SUMO soluble protein allowed us to minimize the toxic effects of the peptide on *E. coli* cells and achieve a higher yield expression. The high purity peptide was finally obtained using chromatographic separation technology. In order to discover the model of LGH2 against pathogenic bacteria, dye leakage, CLSM, SEM and FCM assays were operated to analyze the actions. We found that LGH2 could be bind to cell surface, disrupt cell membrane, and eventually lead to cell death.

## Figures and Tables

**Table 1:** Predicted AMPs from the genome of *L. casei* HZ1.

	Amino Acid Sequence ^1^	Characteristic of AMPs ^2^					
		HR (%)	NC	pI	MW (Da)	HS	BI (kcal/mol)
LGH1	MKHKFARGFFVGTLMTLGAIAGSVFAFKKLYIEPVETKVDEINDNRRKANRKRFSAHQG	34	+4	9.99	3989.47	0.46	4.23
LGH2	MDSQYPKRIFHIIKIWLMIALIALILGLLIGFALGEGNPLKLFLPSTWVHFFKFLR	50	+3	11.17	2447.95	0.55	0.21
LGH3	MQRGMIGAVIGIVLAFAWMRFGFMGMLLVLIFGAAGWLIERYLVPNWHAFTSWLTAGKDAFSKGK	45	+2	9.53	3551.07	0.60	0.76
LGH4	MAAGFIKEVFYSEWFVNSVFVRKKGGKWRMCVDYTGFNKVCSKVFYFLFRID	50	+3	9.24	3266.89	0.48	1.33

^1^ The tentative AMPs were listed with the whole amino acid sequence of the protein; symbol □: conserve Gly-Gly/Ala motif; symbol …: signal peptide; symbol _: mature AMP. ^2^ Boman index (BI) is the protein-binding potential; Hemolytic score (HS) indicates the hemolytic potency, a higher score means a higher hemolytic potency; Hydrophobic rate (HR) is the rate of hydrophobicity amino acid in the sequence of AMP.

**Table 2:** Minimum inhibitory concentrations (MICs) and minimum bactericidal concentrations (MBCs) of LGH2.

Microbial Strains	LGH2		Ampicillin	
	MIC (μM)	MBC (μM)	MIC (μM)	MBC (μM)
*Gram+*				
*Staphylococcus aureus* (ATCC 29213)	4	8	0.25	0.50
*Listeria monocytogenes* (ATCC 7611)	4	8	0.125	0.25
*Lactobacillus casei* HZ1 (our lab)	32	64	0.50	1.00
*Gram-*				
*Klebsiella pneumoniae* (ATCC 700603)	>256	>256	4	8
*Enterobacter aerogenes* (ATCC 13048)	64	128	8	16
*Fungus*				
*Saccharomyces cerevisiae* (our lab)	>256	>256	--	--
*Pichia pastoris* (our lab)	>256	>256	--	--

Note: **--** means no antibacterial effect was detected.

**Table 3:** Percentage of secondary structures according to the deconvolution of CD spectra of LGH2 (100 µM) in water, sodium phosphate buffer, 30 mM SDS, 25% TFE, and 50% TFE.

Solution	α-Helix (%)	β-Sheet (%)	Random Coil (%)
water	4.35	9.66	85.99
10 mM PBS	21.15	9.74	69.11
30 mM SDS	44.32	5.98	49.70
25% TFE	44.69	12.13	43.18
50% TFE	52.76	6.75	40.49

## Supplementary Materials

The following are available online at http://www.mdpi.com/1420-3049/23/9/2246/s1. Figures S1–S10 show RP-HPLC and MS of the chemically synthesized peptides LGH1, LGH2, LGH3, LGH4 and FITC-LGH2, respectively. Figure S11 is a schematic diagram of FITC-LGH2 fluorescein labeling.
