# Supplementary material for: Identification, Recombinant Expression, and Characterization of LGH2, a Novel Antimicrobial Peptide of Lactobacillus casei HZ1
Source: Molecules. 2018 Sep 3;23(9):2246. doi: 10.3390/molecules23092246 (PMC6225214; doi:10.3390/molecules23092246)
Supplement: Supplementary file 1 [file molecules-23-02246-s001.zip › Supplementary 1ú║Molecular mass map of chemically synthesized LGH1 determined by MS.pdf]

Product Name: **LGH1** MW: 3988.60

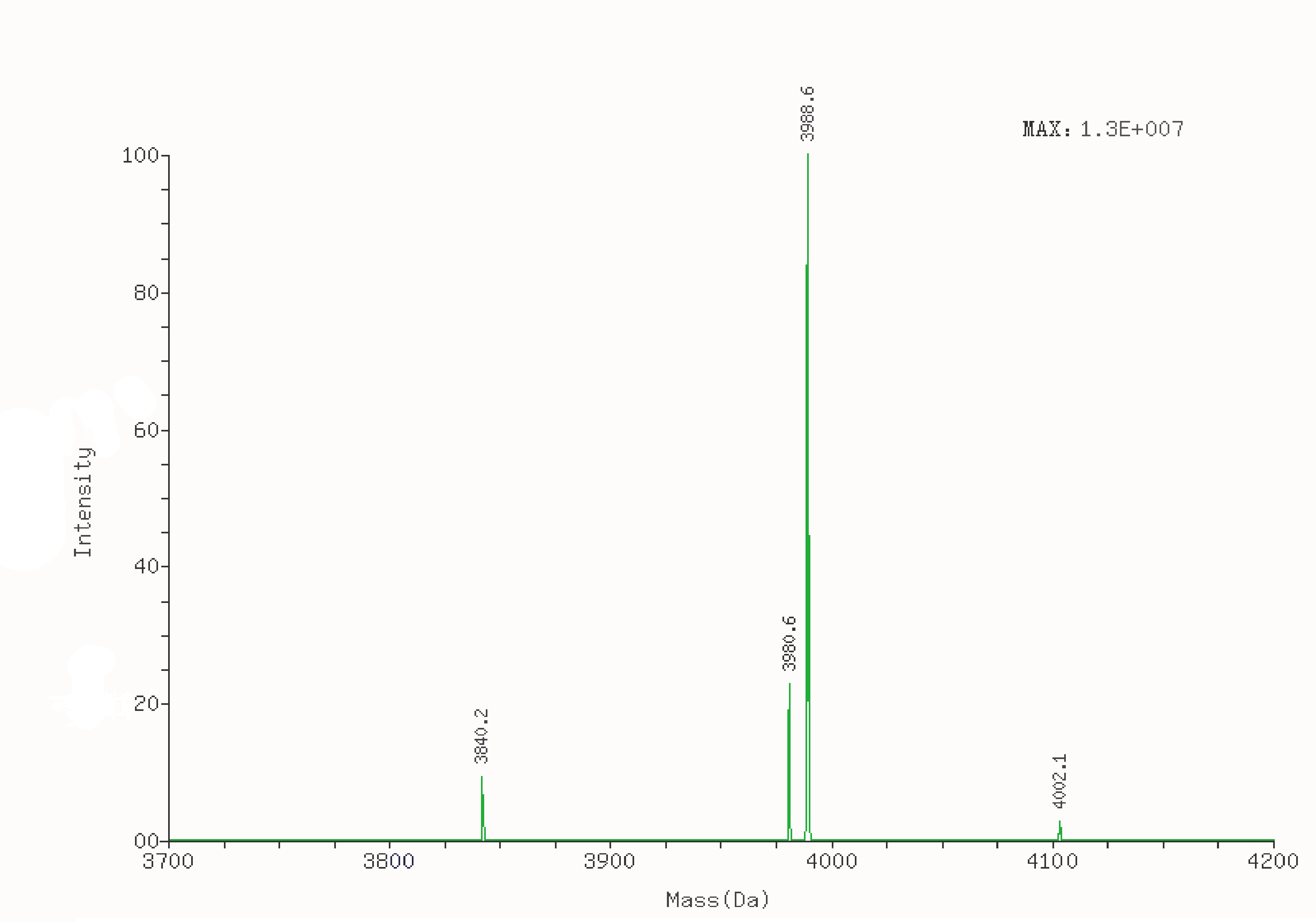

LCQ Deca XP MAX ESI Source

Spray Voltage (kV): 5.02 Spray Current (μA):0.14 Sheath Gas Flow Rate: 35

Aux/Sweep Gas Flow Rate: 0 Capillary Voltage (V):14.85 Capillary Temp (°C): 250.00
