# Supplementary material for: Identification, Recombinant Expression, and Characterization of LGH2, a Novel Antimicrobial Peptide of Lactobacillus casei HZ1
Source: Molecules. 2018 Sep 3;23(9):2246. doi: 10.3390/molecules23092246 (PMC6225214; doi:10.3390/molecules23092246)
Supplement: Supplementary file 1 [file molecules-23-02246-s001.zip › Supplementary 2ú║Purity identification of chemically synthesized LGH1 by HPLC.pdf]

HPLC REPORT

Product Name	:	**LGH1**		
Column	:	VYDAC-C18,4.6*250,5um		
Solvent A	:	0.1%Trifluoroacetic in 100% Water		
Solvent B	:	0.1%Trifluoroacetic in 100% Acetonitrile		
Gradient	:		A	B
		0.0min	80%	20%
		20min	10%	90%
		25min	0%	100%
		30.0min	Stop	
Flow rate	:	1.0ml/min		
Wavelength	:	220nm		
Volume	:	20ul		

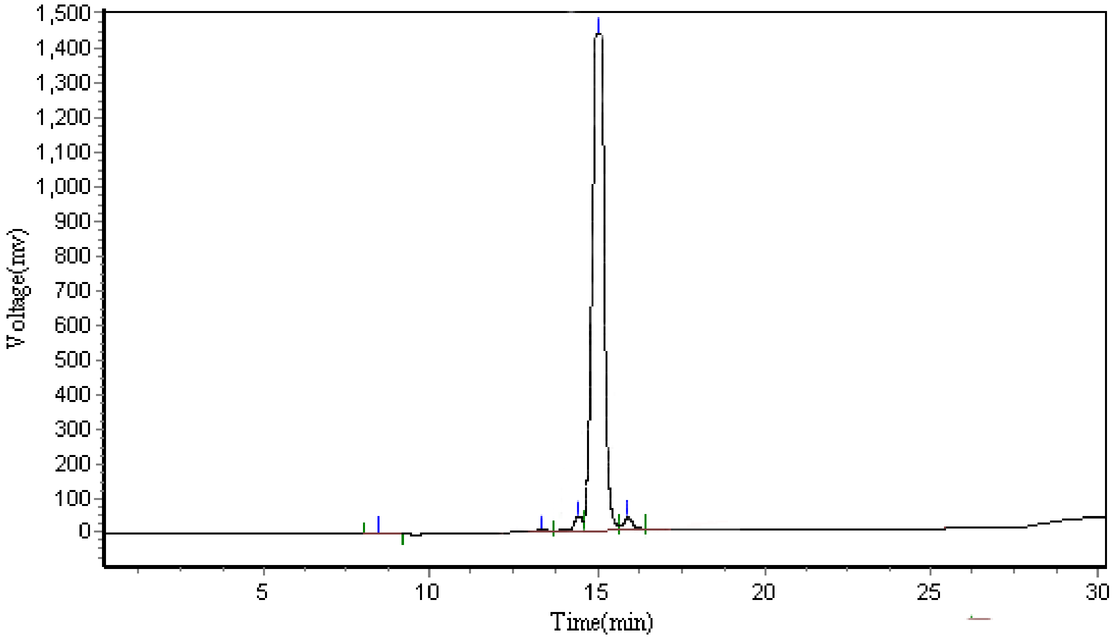

Results

Peak No.	Peak ID	Ret Time	Height	Area	Conc.
1		8.423	1127.764	21368.148	0.0551
2		13.380	5345.105	191592.891	0.4945
3		14.510	40983.762	737259.625	1.9027
4		15.092	1435209.250	36939320.000	95.3337
5		15.958	37689.605	857865.188	2.2140
**Total**			1520355.487	38747405.852	100.0000
