# Supplementary material for: Identification, Recombinant Expression, and Characterization of LGH2, a Novel Antimicrobial Peptide of Lactobacillus casei HZ1
Source: Molecules. 2018 Sep 3;23(9):2246. doi: 10.3390/molecules23092246 (PMC6225214; doi:10.3390/molecules23092246)
Supplement: Supplementary file 1 [file molecules-23-02246-s001.zip › Supplementary 4ú║Purity identification of chemically synthesized LGH2 by HPLC.pdf]

HPLC REPORT

Product Name	:	**LGH2**		
Column	:	VYDAC-C18,4.6*250,5um		
Solvent A	:	0.1%Trifluoroacetic in 100% Water		
Solvent B	:	0.1%Trifluoroacetic in 100% Acetonitrile		
Gradient	:		A	B
		0.0min	80%	20%
		20min	10%	90%
		25min	0%	100%
		30.0min	Stop	
Flow rate	:	1.0ml/min		
Wavelength	:	220nm		
Volume	:	20ul		

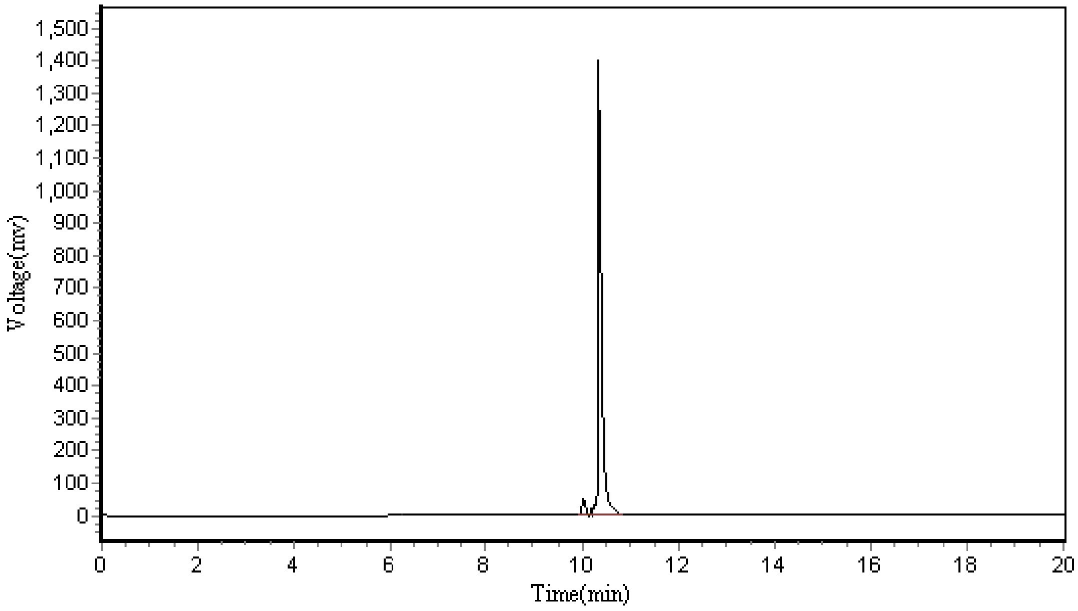

Results

Peak No.	Peak ID	Ret Time	Height	Area	Conc.
1		5.908	8329.248	50915.699	0.6079
2		9.993	42883.574	159455.828	1.9038
3		10.257	3176.064	26585.191	0.3174
4		10.320	1487756.875	8033767.500	95.9204
5		10.953	12256.503	104725.602	1.2504
**Total**			1554402.264	8375449.820	100.0000
