# Supplementary material for: Identification, Recombinant Expression, and Characterization of LGH2, a Novel Antimicrobial Peptide of Lactobacillus casei HZ1
Source: Molecules. 2018 Sep 3;23(9):2246. doi: 10.3390/molecules23092246 (PMC6225214; doi:10.3390/molecules23092246)
Supplement: Supplementary file 1 [file molecules-23-02246-s001.zip › Supplementary 6ú║Purity identification of chemically synthesized LGH3 by HPLC.pdf]

HPLC REPORT

Product Name	:	**LGH3**
Column	:	VYDAC-C18,4.6*250,5um
Solvent A	:	0.1%Trifluoroacetic in 100% Water
Solvent B	:	0.1%Trifluoroacetic in 100% Acetonitrile
Flow rate	:	1.0ml/min
Wavelength	:	220nm
Volume	:	20ul

Gradient :

	A	B
0.0min	80%	20%
20min	10%	90%
25min	0%	100%
30.0min Stop		

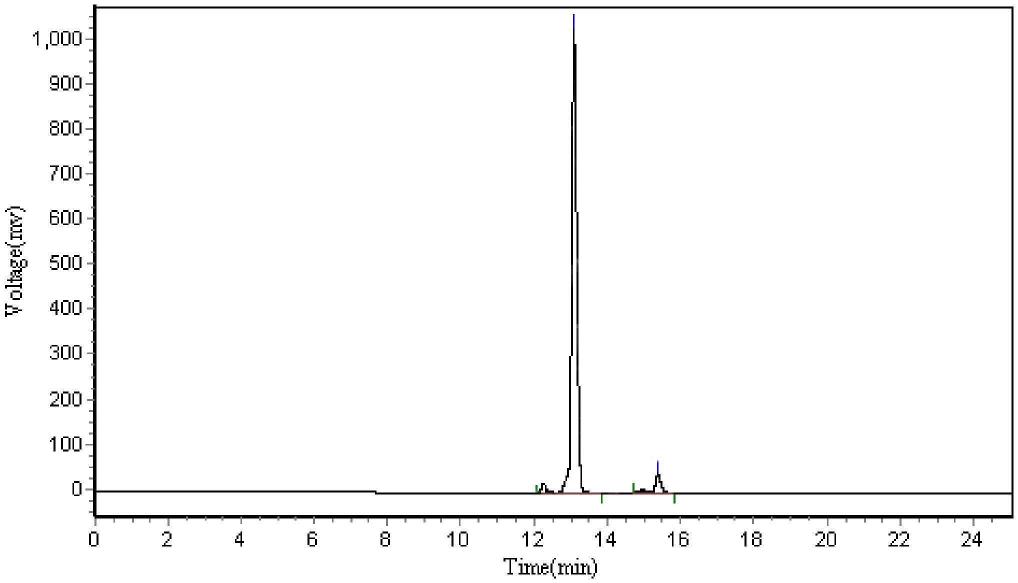

Results

Peak No.	Peak ID	Ret Time	Height	Area	Conc.
1		13.068	1025443.625	10161958.000	95.6654
2		15.868	42572.301	460443.313	4.3346
**Total**			1068015.926	10622401.313	100.0000
