# Supplementary material for: Identification, Recombinant Expression, and Characterization of LGH2, a Novel Antimicrobial Peptide of Lactobacillus casei HZ1
Source: Molecules. 2018 Sep 3;23(9):2246. doi: 10.3390/molecules23092246 (PMC6225214; doi:10.3390/molecules23092246)
Supplement: Supplementary file 1 [file molecules-23-02246-s001.zip › Supplementary 8ú║Purity identification of chemically synthesized LGH4 by HPLC.pdf]

HPLC REPORT

Product Name	:	**LGH4**
Column	:	VYDAC-C18,4.6*250,5um
Solvent A	:	0.1%Trifluoroacetic in 100% Water
Solvent B	:	0.1%Trifluoroacetic in 100% Acetonitrile

Gradient :	A	B
0.0min	80%	20%
20min	10%	90%
25min	0%	100%
30.0min Stop		

Flow rate	:	1.0ml/min
Wavelength	:	220nm
Volume	:	20ul

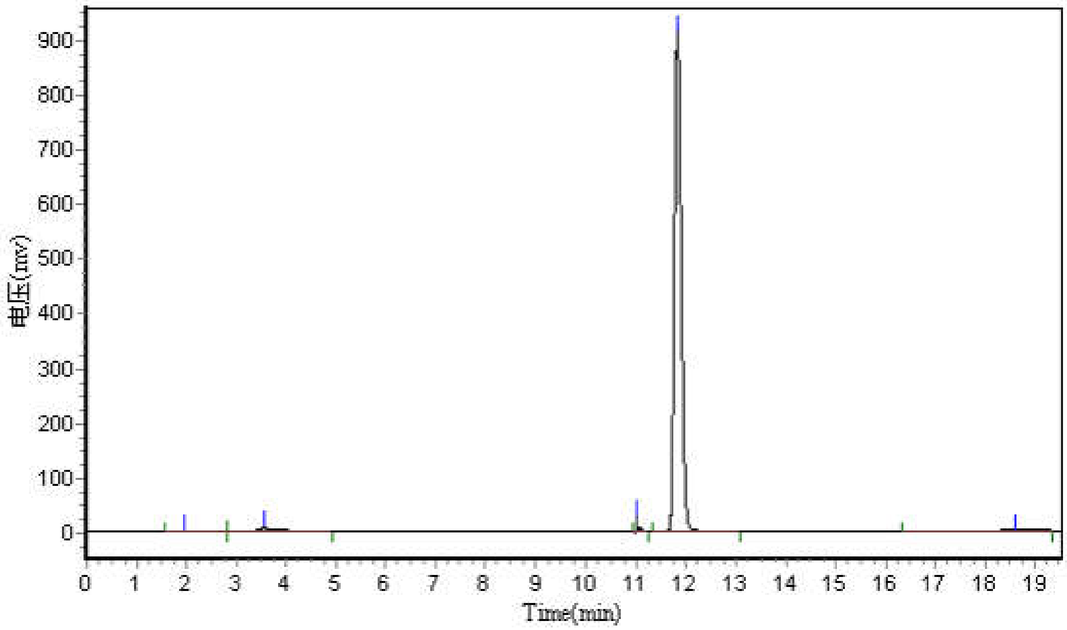

Results

Peak No.	Peak ID	Ret Time	Height	Area	Conc.
1		1.955	3190.085	74238.328	0.8130
2		3.575	6791.360	230544.375	2.5247
3		11.012	30501.564	84741.906	0.9280
4		11.823	908424.250	8693643.000	95.2031
5		18.600	2204.315	48516.750	0.5313
**Total**			951111.575	9131684.359	100.0000
