# Supplementary material for: Identification, Recombinant Expression, and Characterization of LGH2, a Novel Antimicrobial Peptide of Lactobacillus casei HZ1
Source: Molecules. 2018 Sep 3;23(9):2246. doi: 10.3390/molecules23092246 (PMC6225214; doi:10.3390/molecules23092246)
Supplement: Supplementary file 1 [file molecules-23-02246-s001.zip › Supplementary 10ú║Purity identification of chemically synthesized FITC-LGH2 by HPLC.pdf]

# HPLC REPORT

| | | |
|---|---|---|
| Product Name | : | **FITC-LGH2** |
| Column | : | VYDAC-C18,4.6*250,5um |
| Solvent A | : | 0.1%Trifluoroacetic in 100% Water |
| Solvent B | : | 0.1%Trifluoroacetic in 100% Acetonitrile |
| Gradient | : | |

| | A | B |
|---|---|---|
| 0.0min | 80% | 20% |
| 20min | 10% | 90% |
| 25min | 0% | 100% |
| 30.0min | Stop | |

| | | |
|---|---|---|
| Flow rate | : | 1.0ml/min |
| Wavelength | : | 220nm |
| Volume | : | 20ul |

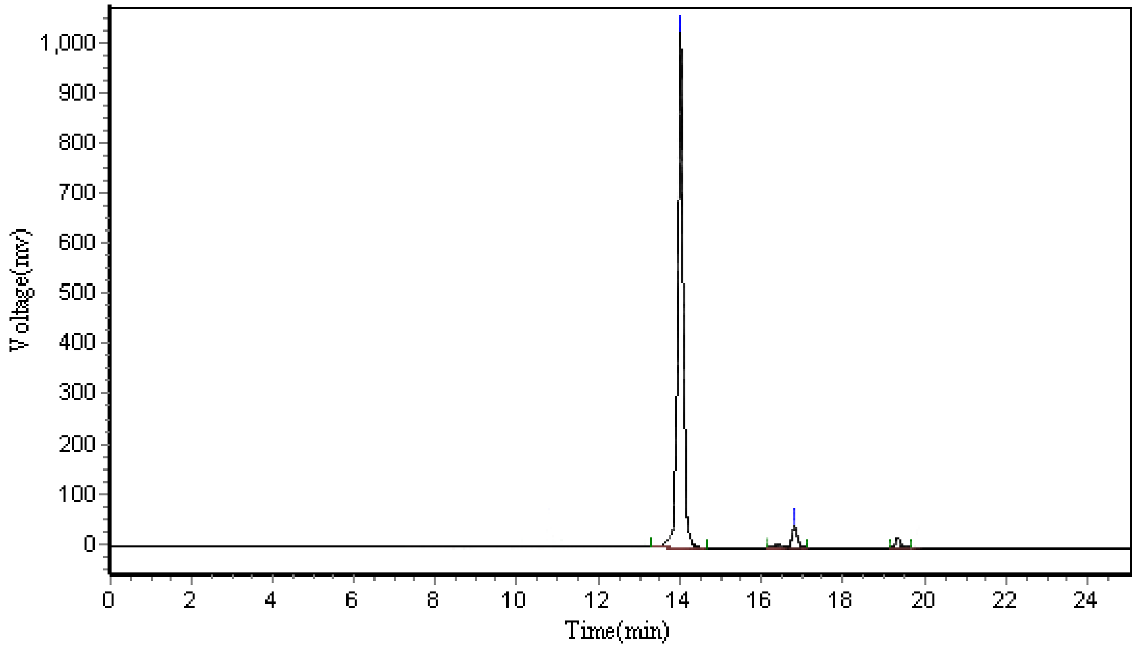


## Results

| Peak No. | Peak ID | Ret Time | Height | Area | Conc. |
|---|---|---|---|---|---|
| 1 | | 14.371 | 1265240.103 | 14211688.409 | 96.0150 |
| 2 | | 16.663 | 31722.609 | 326430.185 | 2.2054 |
| 3 | | 19.325 | 21896.501 | 263413.840 | 1.7796 |
| **Total** | | | 1318859.21 | 14801532.434 | 100.0000 |
