# Supplementary figures and images for: Identification, Recombinant Expression, and Characterization of LGH2, a Novel Antimicrobial Peptide of Lactobacillus casei HZ1
Source: Molecules. 2018 Sep 3;23(9):2246. doi: 10.3390/molecules23092246 (PMC6225214; doi:10.3390/molecules23092246)

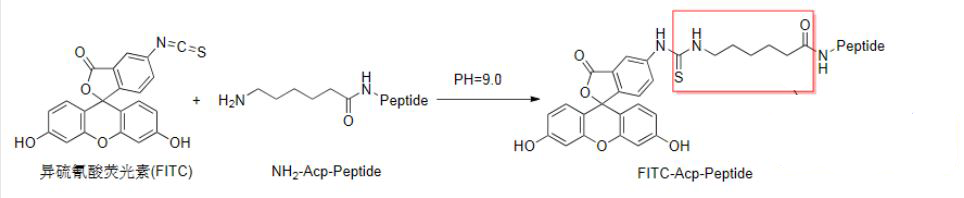

Supplement: Supplementary file 1 [file molecules-23-02246-s001.zip › Supplementary 11ú║Schematic diagram of fluorescent labeling of peptide.tif]
